# A new perspective on intervertebral disc calcification—from bench to bedside

**DOI:** 10.1038/s41413-023-00307-3

**Published:** 2024-01-22

**Authors:** Emanuel J. Novais, Rajkishen Narayanan, Jose A. Canseco, Koen van de Wetering, Christopher K. Kepler, Alan S. Hilibrand, Alexander R. Vaccaro, Makarand V. Risbud

**Affiliations:** 1 Department of Orthopaedic Surgery, Sidney Kimmel Medical College, Thomas Jefferson University, Philadelphia, PA USA; 2 Unidade Local de Saúde do Litoral Alentejano, Orthopedic Department, Santiago do Cacém, Portugal; 3 Rothman Orthopedic Institute at Thomas Jefferson University, Philadelphia, PA USA

**Keywords:** Bone quality and biomechanics, Pathogenesis, Calcium and phosphate metabolic disorders

## Abstract

Disc degeneration primarily contributes to chronic low back and neck pain. Consequently, there is an urgent need to understand the spectrum of disc degeneration phenotypes such as fibrosis, ectopic calcification, herniation, or mixed phenotypes. Amongst these phenotypes, disc calcification is the least studied. Ectopic calcification, by definition, is the pathological mineralization of soft tissues, widely studied in the context of conditions that afflict vasculature, skin, and cartilage. Clinically, disc calcification is associated with poor surgical outcomes and back pain refractory to conservative treatment. It is frequently seen as a consequence of disc aging and progressive degeneration but exhibits unique molecular and morphological characteristics: hypertrophic chondrocyte-like cell differentiation; TNAP, ENPP1, and ANK upregulation; cell death; altered Pi and PPi homeostasis; and local inflammation. Recent studies in mouse models have provided a better understanding of the mechanisms underlying this phenotype. It is essential to recognize that the presentation and nature of mineralization differ between AF, NP, and EP compartments. Moreover, the combination of anatomic location, genetics, and environmental stressors, such as aging or trauma, govern the predisposition to calcification. Lastly, the systemic regulation of calcium and Pi metabolism is less important than the local activity of PPi modulated by the ANK-ENPP1 axis, along with disc cell death and differentiation status. While there is limited understanding of this phenotype, understanding the molecular pathways governing local intervertebral disc calcification may lead to developing disease-modifying drugs and better clinical management of degeneration-related pathologies.

## Back pain and Intervertebral disc degeneration

Chronic low back pain (cLBP) and neck pain are the top causes of years lived with disability and health care costs worldwide.^1,2^ Despite the critical need for clinical approaches to facilitate the social and medical impact, a complete understanding of the etiology and pathophysiology of this painful condition remains elusive.^2,3^ Among several identified factors, disc degeneration is considered a primary contributing factor to cLBP.^3^ Disc degeneration, also described as discarthrosis, has the potential to lead to spine instability, stenosis, spondylolisthesis, and deformity, all described as causes of radiculopathy, chronic back and neck pain, paresthesia, or even neurological deficits.^4–6^ In the US alone, over $100 billion is estimated to be spent annually on direct and indirect costs of disc degeneration and low back/neck pain.^2^ Despite the high socioeconomic burden of disc degeneration, there are no disease-modifiable therapies, and surgery is still the only suboptimal solution for partial symptomatic relief when conservative treatment fails.^7^

The intervertebral disc and adjacent vertebrae comprise a functional motion segment, allowing flexibility, range of motion, and biomechanical properties of the spinal column.^8^ Notably, the interaction between the disc compartments is essential to promote a hydraulic mechanism for handling the loading and mechanical stress intrinsic to spine movement.^9^ The nucleus pulposus (NP) is the central tissue derived from the embryonic notochord and is rich in hydrophilic aggrecan molecules that give the disc its hydration.^10^ The annulus fibrosus (AF) surrounds the NP and restricts the NP swelling with its circumferential and concentric lamellae made of collagen fibers.^11^ Finally, the endplate (EP) comprising a cartilaginous and bony region borders the NP and AF cranially and caudally, allowing the infusion of nutrients and oxygen to cells in these compartments [10].

## Disc degeneration sub-phenotypes

The disc degenerative process is multifactorial and influenced by genetics, obesity, type II diabetes, and lifestyle choices, such as activity status, smoking and nicotine use, and abnormal loading.^12–15^ Moreover, aging is also considered one of the leading risk factors in the progression of disc degeneration and has become more relevant with the increase in life expectancy.^16^ There is ample evidence implicating an essential role of genetics in the predisposition to disc degeneration.^17^ In a twin study, Battié and colleagues showed that genetic background was the top determinant of disc degeneration, surpassing aging and mechanical loading.^18^ Additionally, several single-nucleotide polymorphisms related to matrix anabolism, catabolism, inflammation, and cell signaling have exhibited a strong correlation to disc degeneration.^19–21^ Finally, abnormal spine mechanics in pathologies afflicting the vertebral column, such as scoliosis, kyphosis, and spino-pelvic malalignment, also promote disc degeneration.^22–24^

Irrespective of the etiology, disc degeneration is a heterogeneous pathology and comprises a broad spectrum of phenotypes such as fibrosis, ectopic calcification, herniation, or mixed phenotypes.^5,25,26^ Unlike degenerative pathologies of other related skeletal tissues, the distinction among these phenotypes is often not precise, and there is still some controversy about different stages of degeneration vs. different degenerative phenotypes. Recent animal and human studies suggest that each degenerative phenotype presents a unique molecular and structural signature.^27^ Loss of disc viscoelastic properties characterizes fibrotic phenotypes due to a shift in biosynthesis from proteoglycan rich-matrix towards a stiffer extracellular matrix (ECM) rich in collagens, along with hypertrophic chondrocyte-like differentiation of resident cells and a decrease in disc height.^28–30^ Disc herniation, on the other hand, results from the protrusion of NP content through AF or endplate, with possible neurological symptoms and local inflammation with or without pronounced immune cell ingress.^31^ Disc calcification is possibly the least understood among these phenotypes, usually characterized by calcium phosphate mineral deposition in one or all disc compartments with pronounced cell death.^32^

## Intervertebral disc calcification

Disc calcification is highly prevalent in older individuals and is associated with disc aging^25^ and higher grades of degeneration.^33^ Intriguingly, despite a higher propensity of decline at levels C5/6, T6/7, and L4/5, the distribution of disc calcification throughout the spine exhibits variability among studies.^25,27,34,35^ Whether this process is a response to altered biomechanics or a consequence of a degenerative cascade is still unknown. Additionally, there is an association between disc calcification with systemic diseases such as ochronosis, hemochromatosis, chondrocalcinosis, ankylosing spondylitis, pseudogout, hyperparathyroidism, as well as spine deformities.^25,36,37^ However, the true prevalence of disc calcifications may be underdiagnosed because of the low sensitivity of routine spine imaging techniques such as X-rays and MRI scans.^36,38^ CT scan is the ideal modality for diagnosing and quantifying this phenotype, but no studies have yet characterized the incidence of calcifications seen on CT at the population level.^38^

Histological studies of cadaveric human tissues and animal models suggest disc calcification may occur in all three disc compartments, associated with increased calcium levels, inorganic phosphate, and elevated collagen (COL) 10 and tissue non-specific alkaline phosphatase (TNAP) activity.^27,32^ Interestingly, disc degeneration with a higher prevalence of disc calcification is also linked to increased expression of osterix, RUNX2, bone morphogenic protein (BMP) 2, and osteocalcin (OCN), all known markers of osteogenic differentiation.^27,39,40^ A recent study showed that deficiency of progranulin (PGRN), a pleiotropic growth factor, increases tartrate-resistant acid phosphatase (TRAP), Cathepsin, COL10, matrix metallopeptidase (MMP) 13, and a disintegrin and metalloproteinase with thrombospondin motifs (ADAMTS) 5 levels, leading to dystrophic calcification, disc narrowing, and degeneration in mice.^41^ Disc cells respond to local tissue ossification, but whether they are the initiator of calcification needs to be better established. Interestingly, during pathologies that afflict spinal stability, such as spondylolisthesis, scoliosis, or degenerative progression of the disc, osteophyte formation is usually seen as a physiological response to attain local fusion and, possibly, to promote motion segment stabilization.^42^ Similar to disc degeneration, calcification correlates with the incidence of scoliosis, with prominent endplate involvement, suggesting vertebra-disc co-remodeling to achieve fusion and stability.^32,43^

Unlike intervertebral discs, in large joints, such as the knee, calcium phosphate, and calcium pyrophosphate dihydrate are the most common types of calcium crystals.^44^ Knee meniscus calcification, for example, is found in nearly 20% of the elderly, even without a history of joint disorder. However, 80% of meniscal calcification is associated with cartilage lesions in age-controlled patients.^45,46^ In osteoarthritic menisci, calcification shows a different extracellular matrix composition and higher content of calcium phosphate in comparison to primary chondrocalcinosis, characterized by calcium pyrophosphate dehydrate deposits.^47^ Although the trigger for joint calcification is unknown, there are some well-established hallmarks of the disease: hypertrophic chondrocytes, presenting the high expression of COL10;^39,48^ TNAP, ectonucleotide pyrophosphatase/phosphodiesterase 1 (ENPP1), and progressive ankylosis gene (ANK) upregulation;^49,50^ and local inflammation.^51^ Despite the prevalence of cartilage calcification and its close link to OA and cartilage trauma, the pathophysiology in the knee, similar to the intervertebral disc, is still poorly understood, which hinders the development of disease-modifying drugs.

## Ectopic mineralization-–molecular and cellular pathways

Mineralization, the ordered deposition of hydroxyapatite, is a tightly controlled physiological process restricted to specific locations of the body.^52^ The pathological deposition of hydroxyapatite outside the boundaries of the rigid skeleton is known as ectopic mineralization, a condition that affects millions of people worldwide. Ectopic mineralization is linked to clinical pathologies such as trauma, aging, cancer, diabetes, and autoimmune diseases, all significant causes of morbidity and mortality.^16,52^ Although ectopic mineralization can occur in any tissue, it is often associated with blood vessels, tumors, ligamentous/cartilaginous tissues, and joints.^49,53,54^ Our understanding of how the systems that prevent ectopic mineralization are regulated under physiological conditions still needs to be completed, resulting in an overall lack of success in developing effective treatments for this pathology.

The most common mineral pathologically deposited in soft tissues is hydroxyapatite, composed of calcium phosphate crystals Ca_10_(PO_4_)_6_(OH)_2_. It can, however, also consist of calcium oxalates and octacalcium phosphate, as for instance, often seen in kidney stones.^55^ Two main processes responsible for ectopic calcification are dystrophic and heterotopic ossification, which diverge histologically and in composition.^56,57^ Dystrophic calcification is characterized by amorphous, unorganized calcium phosphate crystals interspersed with necrotic debris, not associated with ECM such as collagens, shown by a high ratio of phosphate to protein content.^27,56,57^ In opposition, heterotopic ossification is characterized by mineralized mature bone, with minerals associated with local ECM, presenting low ratio phosphate to protein, and elevated local activity of TNAP and TRAP associated with osteoblast and osteoclast-like cells.^56,57^

The soft tissues are usually protected against uncontrolled mineralization by two complementary systems: proteins and low molecular weight compounds (<1 000 Da). The first group includes proteins like fetuin-A, osteopontin, and Matrix Gla Protein (MGP).^58^ In contrast, the best-known example of the second group is inorganic pyrophosphate (PPi), which consists of two phosphate groups linked by a hydrolyzable ester bond.^50,53,59–61^ PPi is thought to inhibit mineralization by direct interaction with calcium phosphate ion clusters or nanocrystals, thereby preventing their further growth and keeping them in solution for removal by fetuin-A-dependent mechanisms.^62^ Fetuin-A knockout mice highlight the importance of the interplay between PPi and proteinaceous mineralization inhibitors and do not show an obvious phenotype unless plasma PPi concentrations are substantially decreased.^63^ In contrast, *Mgp*^−/−^ mice show extensive vascular and cartilage calcifications without reduced PPi levels, but the phenotypes are rescued when plasma levels of inorganic phosphate (Pi) are reduced.^64^ Interestingly, in addition to the direct interaction with calcium phosphate clusters, a recent study suggests that PPi may partly exert its biological effects by signaling through G-protein coupled receptor (GPCR) in osteoblasts and osteoclasts.^65^ It is also important to note that PPi is not the only endogenous small molecule in the anti-mineralization network protecting soft connective tissues against pathological deposition of calcium phosphates. Other molecules involved in the modulation of ectopic mineralization include magnesium and citrate.^63,66–68^

**Fig. 1:**
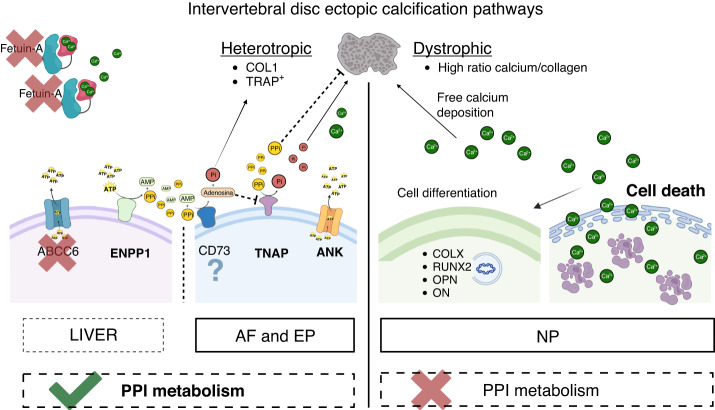
Schematic showing intradiscal calcification pathways. AF and EP present a heterotopic ectopic calcification nature – modulated by PPi metabolism - vs. NP, which shows a dystrophic nature, blinded to PPi metabolism changes. Bold proteins and lines – promote calcification. Dotted lines – inhibit calcification. Cross – no role in intradiscal calcification. Question mark – pathway still not explored in the disc. Proteins involved in extracellular PPi homeostasis. All PPi detected in the circulation originate from ENPP1-mediated conversion of extracellular ATP into AMP and PPi. The two main proteins involved in cellular ATP release for ENPP1-mediated PPi formation in plasma are ABCC6, primarily expressed in the liver, and ANK, ubiquitous. PPi is hydrolyzed to inorganic phosphate (Pi) in the periphery by the ecto-enzyme Tissue Non-specific Alkaline Phosphatase (TNAP). CD73 converts AMP into Pi and adenosine. The latter factor inhibits TNAP activity and, subsequently, PPi degradation. Although ENPP1 is only shown in the liver, this ecto-enzyme is ubiquitously expressed and released into blood plasma

All plasma PPi is derived from adenosine triphosphate (ATP) and other nucleoside triphosphates released into circulation and converted into adenosine monophosphate (AMP) and PPi by ENPP1.^69^ The two most essential proteins mediating the cellular efflux of ATP underlying PPi formation in plasma are ATP binding cassette subfamily C member 6 (ABCC6) and ANK. ABCC6-dependent ATP release from hepatocytes is responsible for approximately 60%–70% of plasma PPi, with the more ubiquitous membrane protein ANK contributing another 20% by sustaining the cellular efflux of ATP from peripheral tissues.^70–73^ Importantly, this multi-pass transmembrane protein ANK provides much of the extracellular ATP needed to form PPi in poorly perfused tissues in addition to mediating the release of citrate and succinate, with the former directly inhibiting mineralization while the latter interacting with specific receptors on the plasma membrane.^73^ In bones and teeth, PPi is hydrolyzed into Pi by phosphatases, of which TNAP is the most prominent.^71^ An additional layer of regulation is afforded by the ectonucleotidase CD73, which cleaves AMP into Pi and adenosine.^74^ The latter inhibits TNAP and lowers PPi degradation. An overview of the factors currently known to participate in extracellular PPi homeostasis is shown in Fig. 1.

ENPP1 plays a central role in PPi generation from ATP. A complete loss of ENPP1 activity results in Generalized Arterial Calcification of Infancy (GACI), a rare condition that, in many cases, leads to death shortly after birth due to massive calcification of the major arteries.^49^ GACI patients have virtually no PPi in their plasma, which explains their severe and acute form of vascular calcification.^75^ On the other hand, Pseudoxanthoma elasticum (PXE) is a milder ectopic mineralization disorder caused by mutations in ABCC6, with clinical manifestations noted in various tissues.^70^ Considering the vital contribution of ANK in PPi homeostasis in tissues that are not well perfused, it is not surprising that loss-of-function mutations in ANK predominantly result in calcification of joint space, articular cartilage, and the disc, eventually resulting in a severe form of ankylosis.^76,77^ Likewise, inactivating mutations in CD73 result in late-onset ectopic mineralization of periarticular ligaments and the arteries of the lower extremities.^78,79^ Finally, the absence of functional TNAP results in hypophosphatasia due to increased local levels of PPi and, consequently, hypomineralization of bones.^80^

In addition to these molecular regulators, several cellular processes participate in pathological tissue mineralization. Inflammation, usually seen in degenerative processes, can trans-differentiate soft tissue cell types such as smooth muscle cells, tenocytes, endothelial cells, and chondrocytes into cells exhibiting osteoblast-like characteristics.^49,81–83^ Extracellular vesicles (EV) secreted by these cells are associated with pathological calcification. In fact, during homeostasis, EV usually contains MGP and fetuin-A, which inhibit local calcification. However, cellular stress causes a shift of EV enriched with calcification makers and calcium phosphate crystals, which promote local calcification by inducing osteogenic and proinflammatory genetic programs and TNAP activity.^84–86^

Similarly, endoplasmic stress response (ER) and autophagy prevent ectopic calcification by promoting cell viability, controlling the secretion of healthy ECM, and facilitating proper protein turnover and intracellular calcium regulation.^87,88^ For example, vascular calcification shows the involvement of activated PERK-eIF2-ATF4-CHOP complex in different mouse models, which further promotes osteogenic differentiation and mineralization through BMP and RUNX2 pathways.^87,89,90^ Similarly, autophagy, a key recycling mechanism active during physiological stress, plays a protective role in the presence of high concentrations of Pi by suppressing mammalian target of rapamycin (mTOR) signaling, counteracting reactive oxygen species (ROS), and protecting against apoptosis, ameliorating ectopic calcification.^91,92^ Finally, cellular fate modulates dystrophic calcification processes. Apoptosis significantly contributes to ectopic calcification by promoting the generation of local calcium phosphate deposits and diminishing the local total capacity of Pi uptake.^93,94^ On the other hand, cellular differentiation is common in ectopic calcification, characterized by upregulation of BMP2, TNAP, and RUNX2.^33,84,95,96^

## Disc calcification: insight from animal models

The most common animal models used in intervertebral disc biology are bovine, dogs, rabbits, rats, and murine (Tables 1–2). Despite the apparent differences, namely the persistence of notochordal-NP cells, quadrupedal, additional vertebrae, and some variations in the extracellular matrix composition, their contribution to understanding molecular and cellular mechanisms of disc degeneration is well accepted.^97^ In fact, due to relatively low cost, availability of various molecular genetics tools, and biological similarities with the human skeleton, mouse models have successfully helped to study and understand several common and rare human conditions and diseases that afflict the skeleton.^60,98–100^ Concerning intervertebral disc, for example, the mouse models have been instrumental in our understanding of the embryonic development and nature of cells within each of the disc compartments.^101–104^ Studies have shown that, despite differences in gait and posture, the distribution of loads on the vertebrae is similar between quadrupeds and bipeds.^105^ Indeed, the orientation of vertebral trabeculae is identical between humans and mice, suggesting a similar loading axis.^105^ While these models need careful interpretation of the findings when drawing parallels to human pathology, they have become valuable tools for understanding various disc degeneration phenotypes, including dystrophic calcification, and even exploring potential therapeutical approaches.^27,106^ Recent advances in histological methods, scoring systems, and the increased availability of genetically modified mice have further contributed to new insights into pathological intervertebral disc calcification (Table 1).^107–109^

**Table 1:** Mouse models that provide insights into the mechanisms underlying intradiscal calcification

Model	Phenotype/insight	Reference
LG/J	**Compartments:** NP + AF + EP **Nature:** Dystrophic **Features/hypothesis:** Increase apoptosis / Calcium deposition without matrix nodules	Novais et al.^27^ Rai et al.^113^
SM/J	**Compartments:** None **Nature:** None **Features/hypothesis:** Systemic absence of fetuin (calcification inhibitor), and high levels of TNAP (calcification promoter)	Novais et al. ^27^ Schäfer et al.^61^
*Sparc*^−/−^	**Compartments:** Endplate **Nature:** Heterotopic **Features/hypothesis:** Decrease Col1 expression / altered mechanical loading	Gruber et al.^130^
*Bmal1*^−/−^	**Compartments:** AF. **Nature:** Heterotopic **Features/hypothesis:** Increased local inflammation / TGF/BMP signaling	Dudek et al.^119^
*ENT1*^−/−^	**Compartments:** AF + Endplate **Nature:** Heterotopic **Features/hypothesis:** decrease extracellular adenosine triphosphate (ATP), and consequently, the production of PPi and adenosine monophosphate (AMP) / no inflammation driven / TNAP increase activity	Warraich et al.^60^ Li et al.^127^
*Abcc6*^−/−^	**Compartments:** None **Nature:** None **Features/hypothesis:** decrease systemic extracellular ATP, which can be converted to AMP and PPi (calcification inhibitors)	Boneski et al.^124^
*ANK*^−/−^ *Ank*^*ank/ank*^ or *ank*	**Compartments:** inner AF + outer AF + EP **Nature:** Dystrophic + Heterotopic **Features/hypothesis:** Increase apoptosis, and inflammation / Autophagy disruption / Cell differentiation with an increase in TNAP/TRAP activity, and COLX and OCN expression	Ohnishi et al.^77^
ENPP1^asj-2j^	**Compartments:** AF + EP **Nature:** Dystrophic + Heterotopic **Features/hypothesis:** Increase apoptosis, and inflammation / Autophagy disruption / Cell differentiation with an increase in TNAP/TRAP activity, and COLX and OCN expression	Zhang et al.^49^

**Table 2:** Phenotypical features of intradiscal calcification seen in non-murine spinal models and their similarity with human pathology

Model	Phenotype/insight	Reference
Sand rats	**Compartments:** EP **Nature:** Heterotopic **Features/hypothesis:** Aging	Gruber et al.^141^
Chondrodystrophic Dogs	**Compartments:** NP and EP **Nature:** Dystrophic **Features/hypothesis:** FGF4 retrogene insertion on CFA12 and 18 leading to increased FGF signaling, aging driven – cell death	Brown et al.^140^ Zhang et al.^166^ Lappalainen et al.^167^ Mogensen et al.^168^
Ovine	**Compartments:** NP, AF, and EP **Nature:** Dystrophic **Features/hypothesis:** Aging/Increase in local/higher prevalence in lumbar – biomechanics/no AP or TRAP activity in the NP compartment/increase in COLX, osteopontin, and osteonectin levels	Melrose et al.^139^
Human	**Compartments:** NP, AF, and EP **Nature:** Heterotopic + Dystrophic **Features/hypothesis:** Aging/Increased local cell death/higher prevalence in lower thoracic – biomechanics/decreased proteoglycan in the disc	Chanchairujira et al.^25^ Hristova et al.^32^ Fournier et al.^56^

## Mouse models of disc calcification

### LG/J and SM/J inbred strains

The link between genetic background and age-dependent progression of disc degeneration in mice has recently been explored in a study utilizing three different inbred mouse strains, C57BL/6, LG/J, and SM/J, which showed distinct degenerative phenotypes, namely, fibrosis, disc calcification, and apoptosis with cell fate change, respectively.^27^ The idea that genetic makeup is an essential contributor to degenerative sub-phenotypes is also supported by twin cohort studies, which showed the strongest correlation between familiar aggregation and disc degeneration, followed by aging and mechanical loading.^17,18^

We recently studied LG/J and SM/J, super and poor cartilage healing mouse strains with similar ancestry, to investigate age-dependent disc degeneration due to their opposing regenerative phenotypes. C57BL6, one of the most widely used mouse strains in research, was also used for comparison.^110–112^ While SM/J mice showed early disc degeneration, starting at eight weeks, LG/J and C57BL6 showed a healthier phenotype at skeletal maturity.^27,110^ However, around 18 months of age, unlike C57BL6 and SM/J strains, LG/J mice exhibited intradiscal calcifications in the caudal spine, which became increasingly prevalent at 23 months, with 80% of studied discs evidencing calcification. Interestingly, despite better cartilage healing potential, LG/J is also shown to have a higher predisposition of synovial and meniscus calcifications following knee trauma,^113^ which further supports a hypothesis that abnormal systemic response to stress, such as aging and trauma, on LG/J genetic background promotes ectopic calcification.

While we observed that LG/J mice possessed widespread calcifications in the NP and AF, no calcification of spinal ligaments was noted, as is seen during ankylosing spondylitis.^60^ Additionally, elevated levels of free calcium, not associated with mineralization nodules or collagen, and increased focal apoptosis suggested the dystrophic nature of this mineralization. Notably, the calcification was restricted to the caudal spine, with a higher incidence seen at Ca6-7, Ca7-8, and Ca8-9, suggesting that crosstalk between anatomic location, local mechanical environment, and genetic background is essential for the predisposition of disc calcification.^34^ While LG/J mice showed normal systemic levels of free Ca^2+^ and phosphorous, TNAP levels were elevated with aging in both LG/J and SM/J mice.^53^ Surprisingly, unlike LG/J mice, SM/J mice did not show any signs of disc calcification despite very low levels of fetuin-A, a known calcification inhibitor, and high levels of TNAP, a promoter of calcification.^61^ These findings suggested that systemic changes in regulators of mineral metabolism alone have little effect on intradiscal calcification.

Mechanistic, transcriptomic, and protein analyses of these three strains suggested a contribution of cell death to elevated free local calcium and phosphate to AF tissue mineralization.^114^ Additionally, resident cells showed an altered differentiation program characterized by increased expression of endochondral genes *Bmp6*, *Tgfb2*, *Runx2*, *Fgf2*, and *Postn* with a decrease in terminal osteoblastic differentiation markers *Sp7*, *Bglap*, and *Alpl*.^115^ An increase in macrophage markers CD14, CD68, and CD163 was also noted, which strongly links macrophage activation with dystrophic calcification in LG/J mice.^116^ Before leaving this topic, it is essential to note that similar to enriched pathways in old LG/J mice, a subset of human NP samples showed an enrichment of genes in pathways concerning response to stress, wound healing, cell death, endochondral bone, inflammation, cell division, phosphorous metabolism, and extracellular matrix organization.

### Mouse models with altered PPi metabolism

ANK is a biological inhibitor of calcified mineralization. Mutations of *Ank* gene in mice promote abnormal mineral deposition in joints and eventually fusion.^76,117^ In humans, the loss-of and gain-of-function mutations in *Ank* are associated with craniometaphyseal dysplasia, ankylosis, and familial chondrocalcinosis, respectively, which are characterized by abnormal facial and long bone growth, skeletal fusions affecting spinal and small articular joints, and hearing ossicles, and in case of chondrocalcinosis, chronic arthropathy resulting from calcium pyrophosphate dihydrate (CPPD) crystal deposition.^118^

*Ank/Ank* mice, which carry a nonsense mutation in exon 11 of the murine ortholog *Ank* (p.E440X), resulting in the C-terminal truncation of the ANK protein by 53 aa and thus a complete loss of ANK function, show severe skeletal phenotypes and disc calcification^76,77^ The discs show altered extracellular matrix composition with decreased abundance of COLI and COMP but increased COL10.^77^ Together with upregulated COLX, there are no changes in MMP13 levels, which indicates that AF cells’ differentiation into a true hypertrophic chondrocyte-like phenotype is unlikely. Noteworthy, the mineralization process observed in the intermediate AF and outer AF is different regarding mechanism, composition, and local cell survival. The intermediate AF was acellular, with high radio-opacity and peripheral cells undergoing apoptosis, suggesting a dystrophic calcium deposition. The outer region presented similar X-ray density to bone and densely residing cells, indicative of a more organized mineralized process. Additionally, the outer cell showed TNAP and TRAP activity, with OCN staining without evidence of monocytes, macrophages, and osteoclasts, suggesting the acquisition of an osteoblastic-like phenotype by resident cells of the AF in *ank* mice.^77^ These results indicate that different tissues and zones may present a divergent cellular and possibly signaling response to local PPi and calcium deposition, ultimately determining the local mineralization process and nature.^65^

Noteworthy, transcriptomic analysis highlighted the consequences of *Ank* loss in affecting ectopic calcification-linked pathways, such as autophagy, with downregulating genes related to protein homeostasis and heat shock protein suggesting proteostasis.^92^ Additionally, we observed Basic Helix-Loop-Helix ARNT Like 1 (BMAL1)/ Clock circadian regulator (CLOCK) dysregulation in the NP and AF in *ank* mice, involving several key regulators of the circadian clock – *Cry2*, *Nr1d1*, and *Per3*. Like *ank* mice, *Bmal1*^−/−^ mice show disc calcification in the AF compartment and cortical and trabecular bone loss.^77,119,120^ These results suggest that different tissues may show a divergent cellular response to changes in local PPi levels and calcium deposition, ultimately determining the local mineralization status. Importantly, in addition to controlling local PPi and citrate levels ANK may play a central role in disc calcification by modulating BMAL1/CLOCK pathways, cell fate, autophagy, local inflammation, and matrix remodeling which, are known promoters of ectopic calcification.

Another key regulator of local PPi levels is ENPP1, an enzyme that hydrolyzes ATP to AMP and PPi.^121^ Asj-2J mice with a spontaneous mutation of ENPP1 have been used as a model of generalized arterial calcification of infancy (GACI). These mice show ectopic calcification in several tissues, including ears, muzzle skin, trachea, aorta, and vertebrae.^121^ Ectopic calcification in asj-2J mice was only noted in the CEP and AF compartments, without associated osteoblastic and osteoclastic activity, as early as 4–11 weeks of age.^121^ Together with the phenotype seen in *ank* mice, the results indicate that PPi levels in AF and CEP compartments are critical to prevent ectopic calcification in the disc.^77,121^ Considering the dystopic nature of the calcification processes and the presence of these phenotypes only in collagen-rich tissues, we speculate that the higher content of proteoglycans and lower levels of collagens characteristic of the NP compartment may be protective for calcium phosphate deposition, even under conditions of low PPi levels.^77,121,122^

Similar to ANK, ABCC6 inhibits ectopic mineralization by regulating circulating levels of extracellular ATP, which is converted to AMP and PPi by ENPP1.^70^ The loss-of-function mutations of ABCC6 cause pseudoxanthoma elasticum (PXE), an autosomal recessive metabolic disorder characterized by ectopic mineralization in elastin-rich tissues.^123^ Despite ectopic calcification being seen in *Abcc6*^−/−^ mice in elastin- and collagen-rich connective tissues such as eye, skin, and arteries along with reduced vertebral bone quality, there was no evidence of calcification in the disc.^124^ It is plausible that the avascular environment of the disc is less sensitive to systemic changes in calcium and PPi levels, a conclusion also supported by the phenotypic assessment of *ank*/*ank*, LG/J, and SM/J mice. Furthermore, the unique ECM composition, containing cartilage oligomeric matrix protein (COMP) and proteoglycan aggrecan, known inhibitors of calcification,^54,125^ may be sufficient to prevent disc mineralization in *Abcc6*^−/−^ mice.

### *Slc29a1*^−/−^ mice

Null mutation of the *Slc29a1* locus, which encodes equilibrative nucleoside transporter1 (ENT1) – a transmembrane transporter of hydrophilic nucleosides, such as adenosine – associates with ectopic mineralization of the spine in humans.^126^ Similarly, *ENT1*^−/−^ mice develop ectopic mineralization of the fibrous connective tissues of the spine, including the AF and adjacent ligaments.^60^ Consequently, these mice have been successfully used as a model of Diffuse Idiopathic Skeletal Hyperostosis (DISH), a noninflammatory spondyloarthropathy characterized by ectopic calcification of spinal tissues.^60,127^ Noteworthy, *ENT1*^−/−^ mice follow the spatial, temporal, and anatomical ectopic mineralization pattern seen in humans with DISH.^60^ It is important to note that calcification is first observed in *ENT1*^−/−^ mice between 6 and 8 weeks of age in the paraspinal connective tissues of the cervical vertebrae, developing gradually and caudally until becoming symptomatic by 8 months.

Interestingly, this process occurs without inflammation and, as expected, with significantly elevated plasma adenosine levels. Of note, expression of *Mgp*, *Enpp1*, *Ank*, and *Spp1*^113,128^ were significantly downregulated in the discs of ENT1 null mice. This suggests a crosstalk between ENT1, ENPP1, and ANK in AF cells, further supporting their importance in inhibiting disc mineralization. The authors of this study noted that the expression of *Alpl*, responsible for the hydrolysis PPi, was decreased in the *ENT1*^−/−^ mice and interpreted this finding as a compensatory down-regulation in response to ectopic mineralization within the disc. However, we opine that this observation could reflect increased plasma adenosine levels, as adenosine is known to inhibit *Alpl* expression. Surprisingly, however, in contrast to mRNA levels, micro mass cultures of AF cells from *ENT1*^−/−^ mice under conditions that promote mineralization showed elevated TNAP activity, an observation further supported by higher TNAP activity staining in discs in situ, suggesting a possible local source of mineralization and higher tissue-specific potential to mineralize.^127^

### *Sparc*^−/−^ mice

Secreted protein, acidic and rich in cysteine (SPARC), also known as osteonectin, is a glycoprotein secreted by osteoblasts during mineralization that binds to calcium and promotes crystal formation.^129^ Interestingly, SPARC levels decrease during disc degeneration and aging.^130^ To follow-up on this, Gruber and coworkers first described the disc phenotype of *Sparc* mutants, which showed accelerated disc degeneration with increased lumbar herniations and endplate calcification and sclerosis.^130^ Additionally, *Sparc* null mice develop chronic back pain, demonstrating similar behavioral radiculopathy symptoms observed in humans.^131^ While disc herniation is directly linked to radiculopathy, endplate changes including calcification and sclerosis also promote chronic back pain.^35^ Notably, spinal hypersensitivity seen during aging or lumbar spine instability is promoted by osteoclast-dependent sensory innervation in porous areas of the sclerotic endplates.^132^ Accordingly, inhibiting osteoclastic activity or formation by deletion of *Netrin1* in osteoclasts or of *Rankl* in the osteocytes, respectively, prevents sensory innervation and hypersensitivity.^131^ Likewise, increasing osteogenesis in sclerotic, porous endplates of mice with lumbar spine instability by resveratrol treatment leads to decreased hyperalgesia.^133^ SPARC is also known to induce the expression of metalloproteases and collagen, which may affect local tissue homeostasis.^134^ *Sparc* knockdown decreased the collagen I expression by AF cells, which may explain the susceptibility to disc herniation in *Sparc* null mice.^129^ Significantly, changes in the disc ECM are associated with altered mechanical loading, leading to endplate calcification.^35^ However, possible dysfunction of endplate cells caused by *Sparc* modulation may also be involved in this process, as shown in aortic ectopic calcification, by disrupting BMPR-II/p-p38 signaling.^135^ Overall, these studies underscore the uniqueness of each disc tissue regarding the mineralization process.

### *Bmal1*^−/−^ mice

The intervertebral disc function depends on the circadian clock to maintain tissue homeostasis.^119,136^ Mutations in *Bmal1*, a core clock gene, accelerate disc degeneration and modulate the local inflammatory state.^119^ *Bmal1*^−/−^ mice show ectopic calcification restricted to AF, suggesting divergent functions of this pathway across disc tissues. Notably, disruption of BMAL1 also promotes heterotopic calcification of tendons and ligaments with aging via activation of TGF/BMP signaling.^137^ The functional and structural similarities between AF and tendons/ligaments suggest the critical role of BMAL1 in preventing calcification of ligamentous and fibrocartilaginous tissues. Consequently, during aging, dysregulation and lower levels of BMAL1 may explain the increase in the prevalence of disc calcification by activating TGF/BMP signaling, as noted in ligamentous tissues.^138^ Supporting this crucial role of *Bmal1* contribution to disc calcification, *ank* mutant mice with dysregulated *BMAL1/CLOCK* signaling showed similar dystrophic mineralization of the AF. Further studies are needed to explore the possible downstream effectors and molecular targets of BMAL1 signaling to prevent this mineralization process.^77,119^

### Non-murine models of disc calcification

There are clear advantages of murine models in studying the molecular regulation of biological processes through genetic and injury manipulation.^97^ Nevertheless, intervertebral disc calcification has been well documented in several non-murine models, including ovine,^139^ chondrodystrophic dogs,^140^ and sand rats (Psammomys obesus) (Table 2).^141^ The sand rats show a prominent EP calcification as one of the main features during the age-dependent progression of disc degeneration.^141^ Melrose et al. described the presence of disc calcification in all three compartments on the ovine discs during aging, with different distribution along the spine, presenting maximal incidence at the lower lumbar level and no deposits in the lumbosacral and lower thoracic disc.^139^ These results further support the contribution of the interplay between the mechanical factors and anatomical location to disc calcification in line with observations in humans and aged LG/J mice.^27,32^ Moreover, the mineral deposits in aged ovine discs were composed of hydroxyapatite, showing larger crystallite size than cortical bone, associated with no COL10, osteopontin, and osteonectin, suggesting a dystrophic process, similar to the aged LG/J mice. Furthermore, proteoglycans, known to inhibit calcification, were lower in the calcified disc, possibly justifying the increased susceptibility of ectopic calcification during disc aging.^27,139^

Genome-wide studies of chondrodystrophic dogs have identified a significant association between disc degeneration, namely with calcification and a polymorphism causing overexpression of FGF4 associated with insertion of FGF4 retrogene on domestic dog (*Canis familiaris*, CFA) chromosome 12 and 18 - CFA12 and CFA18.^140^ However, other models have not explored the molecular basis of this phenomenon. While these models fail to study the causative relation between molecular mechanisms and disc calcification processes, they underscore the evolutionary nature of this pathophysiological process, suggesting that aging-associated discal calcification is conserved across vertebrates.

## Challenges posed by disc calcification in clinical medicine

From a clinical perspective, few studies have systematically explored the consequences of this disc degeneration sub-phenotype on disease progression, treatment options, and prognosis. Prior studies have described the prevalence of calcified intervertebral disc in the general adult population as approximately 5% on chest radiographs and 6% on abdominal radiographs.^142^ While the prevalence is even higher in elderly patients, symptomatic disc calcifications have classically been considered rare, especially in adults. However, disc calcification has now become associated with various disease processes contributing to pain, stiffness, and altered spine biomechanics, ultimately leading to significant patient morbidity.

One of the main challenges in clinical medicine is distinguishing the extent of symptoms caused by a calcified disc versus concurrent pathologies affecting the spine. Broadly, it appears that disc calcification can be grouped into 3 potential pathophysiologic processes: inflammatory, mechanical, or degenerative.^35^ Some of the inflammatory or systemic processes that have been described include ochronosis, hemochromatosis, and chondrocalcinosis. Ochronosis results from an enzyme deficiency leading to excess homogentisic acid deposited in joints and the disc space. It demonstrates some of the most impressive radiographic features for patients later in life, including AF and NP calcification. Hemochromatosis and chondrocalcinosis also have disc calcification, but their appearance is less pronounced on radiography, and much of what we know about their presence in the disc space comes from cadaveric studies.^37^

The two other critical processes associated with calcification, mechanical and degenerative, are manifest in many spinal disease states and have significant overlap. Scoliosis is one of the most common diseases in which we see disc calcification. Scoliosis is a three-dimensional curvature of the spine, resulting in abnormal mechanical loading and deformity if allowed to progress. Hristova et al. discuss how the calcification seen in scoliotic patients occurs more often in the cells of the endplates and how the extent of calcification can differ between convex and concave parts of a curve due to differences in proteoglycan content. Calcium deposits and collagen 10 in intervertebral disc are also present in scoliotic and disc degeneration patients but are absent in control discs. Zehra et al. postulate that these disc architecture and calcification patterns similarities suggest that even adolescent idiopathic scoliosis may be a premature degenerative process.^35^ While abnormal loading mechanics are characteristic of scoliotic discs, there remains no clinical consensus on whether the disc calcification in these patients contributed to or was a result of their pathology. Regardless, disc calcification has also been shown to predict spine flexibility and segment instability and should be further studied for its potential role in deformity.^143,144^

Prior case reports have associated disc calcification with discogenic back pain, one of the most challenging diagnoses to manage, even without calcification. Nogueira-Barbosa and Azizaddini et al. present acute thoracolumbar and cervical pain cases, respectively, in patients who had a calcified disc in the location of their pain without any neurologic deficits.^142,145^ Both cases involved middle-aged patients with unremarkable symptom onset and were managed nonoperatively with pain medication and physical therapy. Interestingly, in both cases, the calcification reached complete resolution after follow-up, with the cervical study documenting radiographic disappearance up to 6 months after symptom onset.

**Fig. 2:**
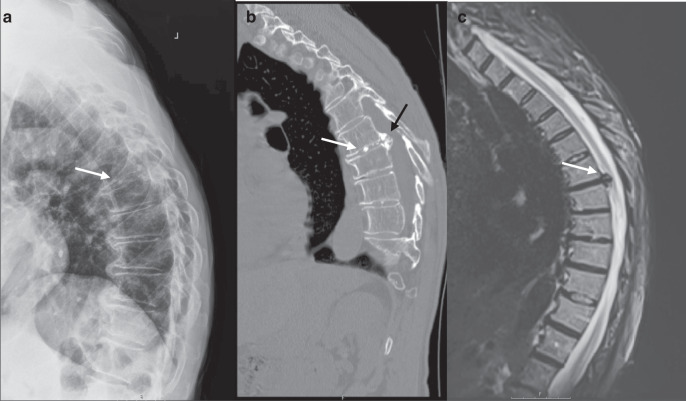
Clinical presentation of disc calcification - X-ray and MRI images. 72-year-old male who presented with a calcified thoracic disc herniation for pre-surgical workup. **a** Preoperative lateral view x-ray demonstrating intervertebral disc calcification (arrow) in the thoracic spine. **b** CT sagittal reconstruction of thoracic spine demonstrating disc calcification (white arrow) and herniation of calcified material extending posteriorly (black arrow). **c** Sagittal T2-weighted MRI demonstrating disc herniation into the spinal canal (white arrow); calcified material within the disc space is not clearly visualized

Another challenging aspect of treating disc calcification is when it is present with herniation (Fig. 2). There have been several reports on this phenomenon, and it is also one of the few disc calcification presentations with a surgical solution. Calcified herniated discs are still challenging to treat as they are associated with myelopathy and intradural extension.^146^ Even after surgical resection, they can still be related to poor outcomes and complications postoperatively. Depending on their size and location, thoracic calcified disc herniations require unique preoperative planning. Some approaches are performed through the thoracic cavity to achieve adequate resection and decompression of the neural elements.^147,148^

Radiographic and advanced imaging studies have demonstrated that no single imaging study can adequately characterize all disc calcifications (Table 3). Most clinicians will begin with a conventional radiograph, but as discussed, the prevalence of calcifications seen on X-rays in patients with symptoms of pain, stiffness, and spinal deformity can be low. Computed tomography (CT) scans have also been described in numerous studies as a method to confirm disc calcifications.^142,145^ One issue with utilizing CT scans is the higher radiation exposure, which is an important consideration when evaluating pediatric patients. The other concern is that while it may provide detail on the nature and location of calcification, it gives little piece on the spinal cord’s or nerve roots' state. Technetium bone scan was used in a case report that demonstrated increased uptake at the level of a thoracic disc calcification; however, it proved to be non-specific, with the entire vertebral body adjacent to the disc also showing increased uptake.^142^ Magnetic Resonance Imaging (MRI) can indirectly assess the content of the disc through water content; however, there are no clear criteria for distinguishing calcifications. Zehra et al. also discuss that high signal intensity on T1-weighted MRI associated with disc calcification was challenged and may be associated more with fatty infiltration.^36,149,150^ A more recent MRI technique has sparked interest, called ultra-short time-to-echo (UTE) MRI. Specifically, the “UTE Disc Sign,” or UDS, was established by comparing UTE MRI studies to radiographs of patients with IVD calcifications. The study authors further proposed that UTE MRI may be able to identify subtle and unique disc changes that may not be revealed on plain radiographs or conventional MRI.

**Table 3:** Comparison of diagnostic imaging modalities for intravertebral calcification

Imaging modality	Advantages	Disadvantages
Conventional radiograph	• Low cost with good specificity • Standard part of most spine pathology works up	• Lower Specificity; can miss subtle calcifications due to poor image quality/malrotation or small foci of calcific deposits
Computed tomography (CT)	• High specificity, can also provide more detail on quality and location of the calcification focus	• Higher radiation exposure • Unable to properly evaluate neural elements
Conventional magnetic resonance imaging (MRI)	• Can evaluate neural elements • No radiation exposure	• Questionable reliability in detecting IVD calcification
Ultra-short time-to-echo (UTE) MRI	• Allows for greater detail of longitudinal ligaments, annulus fibrosus, and the CEP • The uncalcified and calcified portions of the CEP can be distinguished.	• Limited accessibility: requires high gradient MRI performance and specific software^169^
Technetium bone scan	• Allows for localization of a lesion or abnormality to the vertebral body, disc space	• Disc Space can often be obscured by surrounding uptake^170^

In the context of back pain, disc calcification may be responsible for changes in local mechanics, inflammatory reactions, and accelerating disc degeneration.^35,151^ Furthermore, disc calcification can also predict spine flexibility and segment instability. Knowledge of these associations and advancements in imaging techniques will help determine appropriate indications and allow providers to select the best treatment, be it nonoperative or a variety of surgical approaches (Table 3).^35,144^

## Disc calcification therapeutical solutions

Several approaches have been explored to regenerate and prevent the progression of disc degeneration with minimal success.^152^ A critical aspect of this challenge is the environment of the disc space itself, which has avascular, acidic, and hypoxic properties.^153^ The cartilaginous endplate, which facilitates the diffusion of nutrients, begins to undergo progressive calcification throughout degeneration. This results in worsening nutrient diffusion. Cellular metabolism also shifts towards more anaerobic pathways, further decreasing the pH of the disc. These factors compromise the function of existing NP cells but also act as a barrier to the proliferation of regenerative cells.^154^

Regarding disc calcification, there are no specific therapeutic modalities available yet. While mesenchymal cell transplantation may represent a promising approach to treat degeneration in other joints,^155^ chondrogenic differentiation of cells often leads to the hypertrophic rather than articular phenotype, promoting local mineralization.^156,157^ Recently, *Abcc6*^*−/−*^ mice treated with potassium citrate showed a dose-dependent decrease in TRAP levels and improvement in vertebral bone health parameters. This approach may be used to treat disc mineralization by promoting direct chelation of Ca^2+^ by citrate and the dissolution of intradiscal calcium deposits by inducing metabolic acidosis, as well as ameliorating local mineralization signaling and promoting cell survival.^124,158^

A case report of a 40-year-old man with chronic low back pain due to ochronosis presented a symptomatic decrease in calcium deposition after treatment with anakinra, an IL-1 antagonist drug.^159^ This approach has already been successfully used for treating refractory calcium pyrophosphate deposition-induced (CPPD) arthritis.^160^ In treating dystrophic vascular calcification, a few compounds have shown promising results in delaying or decreasing ectopic calcification, such as bisphosphonates;^161^ Vitamin D^162^ and Vitamin K^163^ and chelating drugs such as EDTA^164^ and citrate.^158^ These agents may hold some promise in treating disc degeneration accompanied by prominent calcification phenotype.

Recombinant human growth and differentiation factor-5 (rhGDF-5) has also been explored in phase 2 clinical trials as an intradiscal therapy for degenerative disc disease, showing promising results (NCT00813813 and NCT01124006). Experiments in rabbit models of disc degeneration showed that intradiscal injections of rhGDF-5 can increase disc height and hydration. Guo et al. have proposed that it can also inhibit calcification of the endplates and annulus fibrosus, thereby further promoting nutrient supply to the disc.^165^ However, clinical results have yet to demonstrate the treatment’s effect on disc calcification.

## Conclusions

Disc calcification is a poorly understood degenerative phenotype and poses a significant challenge in the clinical management of patients. Since the personalized approach to treating spine disease is gaining traction, and with an increase in the aging population, the urgency to better understand the different degenerative sub-phenotypes to optimize the therapeutic approach has never been more pressing. The need for well-accepted animal models poses challenges in understanding the etiology and fundamental mechanisms driving disc calcification. However, based on the recent findings from various animal models, disc calcification appears to be a complex pathophysiology with different presentations according to the disc compartment (Tables 1–2).

These studies indicate that AF and EP are more susceptible to ectopic calcification in scenarios where local PPi homeostasis is compromised.^49,77,127,130^ Interestingly, this process in the AF is usually followed by an increase in local TNAP and TRAP activity and resident cell differentiation into osteoblastic-like phenotype.^77,127^ Altogether, these data show that the calcification of the AF and EP may share similarities with mineralization mechanisms noted in other collagen-rich tissues such as ligaments and skin.^49,52,53,56,77^ On the other hand, systemic modulation of free calcium and PPi do not seem to play an essential role in driving disc calcification.^27,124^ Finally, similar to human patients, NP calcium deposits are only noted in aging models, including mice and sheep. This phenotype is generally associated with decreased proteoglycan levels, increased cell death, and increased levels of COL10.^27,32,139^ While genetic background seems to play an essential role in the susceptibility to disc calcification, we found an increased prevalence of calcification in lumbar and caudal levels in both ovine and mouse models, respectively.^18,27,139^ In human studies, approximately ~6% of degenerated human discs present discal calcification, with a higher prevalence in aged discs and the AF compartment.^25^ Like animals, an increased prevalence in a specific region, namely the lower thoracic, is noted, implying that the interaction of biomechanical factors with aging and genetic predisposition promotes disc mineralization.^25^

In summary, anatomic location, genetic predisposition, and environmental stress, such as aging or trauma, strongly influence intervertebral disc calcification phenotype. Efforts must be made to understand the unique regulation of the intervertebral disc as an organ system to improve the outcome of new treatments in the future.
